# Impact of Maxillary Sinus Septa on Sinus Volume: A Retrospective Cross‐Sectional Radiographic Study

**DOI:** 10.1155/ijod/1769882

**Published:** 2026-02-24

**Authors:** Mohammed Amjed Alsaegh, Wael Nabil Kharoufah, Mhd Omar Al Jouhari, Shishir Ram Shetty, Asok Mathew, Okba Mahmoud

**Affiliations:** ^1^ Department of Oral and Craniofacial Health Sciences, College of Dental Medicine, University of Sharjah, Sharjah, UAE, sharjah.ac.ae; ^2^ Research Institute for Medical and Health Sciences, University of Sharjah, Sharjah, UAE, sharjah.ac.ae; ^3^ Department of Clinical Sciences, College of Dentistry, Ajman University, Ajman, UAE, ajman.ac.ae; ^4^ Center for Medical and Bio-allied Health Sciences Research, Ajman University, Ajman, UAE, ajman.ac.ae

**Keywords:** CBCT, maxillary sinus, scan, septa, volume

## Abstract

**Objectives:**

This study aimed to evaluate maxillary sinus volume and its relationship to maxillary sinus septa using cone‐beam computed tomography (CBCT).

**Materials and Methods:**

This retrospective cross‐sectional study assessed 253 CBCT scans (506 sinuses) of adult patients from the archive of the University Dental Hospital of Sharjah (UDHS) using Planmeca Romexis software. Sinus volume was measured, and septa were assessed for presence, location, orientation, and completeness.

**Results:**

A total of 253 CBCT scans were analyzed, including 116 males (45.8%) and 137 females (54.2%), with a mean patient age of 39.3 ± 13.6 years. These scans encompassed 506 maxillary sinuses assessed for the presence of septa, while 467 sinuses were suitable for volumetric analysis. Septa were present in 48.2% of cases, with nearly equal unilateral and bilateral distributions. Males had significantly larger sinus volumes than females (14.68 ± 5.38 cm^3^ vs. 12.10 ± 3.61 cm^3^; *p*  < 0.001), and no significant differences in sinus volume were observed among age groups (*p* = 0.508). Sinus volume was significantly higher in the presence of septa (*p*  < 0.001), while septa position, orientation, and completeness had no significant impact (*p*  > 0.05). Septa were most commonly located in the middle region (*p*  < 0.001) and were predominantly oriented coronally (*p*  < 0.001). No significant differences in sinus volume or septa prevalence were observed among fully dentate, partially edentulous, and completely edentulous groups (*p*  > 0.05).

**Conclusion:**

Males exhibited larger sinus volumes, while dentition status showed no significant effect. Septa were present in approximately half the cases, most often in the middle region with predominant coronal orientation, and were linked to greater sinus volume. These findings highlight the value of individualized preoperative CBCT assessment for safe implant and sinus floor elevation planning and question the assumption that sinus pneumatization consistently follows tooth loss.

## 1. Introduction

The maxillary sinuses are air‐filled, pyramid‐shaped cavities lined with epithelial tissue, varying in size and shape. Located within the maxillary bones beneath the orbital floor [1], they are of particular relevance to dental professionals due to their proximity to areas commonly involved in oral and maxillofacial procedures. A thorough understanding of their anatomy is essential for minimizing complications in maxillofacial surgeries, planning for dental implant placement, estimating graft volume for sinus lift procedures, and positioning orthodontic mini‐implants.

Development of the maxillary sinus begins around the 10th week of fetal life, with secondary pneumatization extending the sinus into the maxillary bone and reaching an approximate volume of 6–8 cm^3^ at birth [1, 2]. Postnatal growth occurs predominantly in the lateral and inferior directions and continues with dental development, with the sinus attaining an average adult volume of ~15 cm^3^ by 18–20 years of age [1, 3]. The determinants influencing final sinus volume remain debated due to the challenges in evaluating growth in individuals with anatomically normal facial structures.

Maxillary sinus septa are thin bony partitions projecting from the sinus walls that may partially divide the sinus into compartments [4]. First described by Underwood [5] in 1910 and often referred to as “Underwood septa”, they are classified into primary and secondary types [6].

Primary septa develop during maxillary growth and are more prominent in dentate regions, while secondary septa are believed to result from irregular pneumatization, often occurring in edentulous areas [7]. These structures help support masticatory forces but may regress following tooth loss [8]. Underwood [5] observed that septa commonly recur in specific locations: anteriorly, between the second premolar and first molar; in the middle, between the first and second molars; and posteriorly, near the third molars. Septa are clinically important in sinus lift procedures, as their presence may require modifications to the lateral window design to prevent septal fracture and reduce the risk of Schneiderian membrane perforation.

Although sinus anatomy is well‐studied, limited evidence exists regarding the influence of sinus septa on maxillary sinus volume. This study aimed to investigate maxillary sinus volume and its relationship to sinus septa using cone‐beam computed tomography (CBCT) analysis. This study hypothesized that anatomical variations of the maxillary sinus, including the presence and characteristics of sinus septa, may be associated with differences in maxillary sinus volume.

## 2. Materials and Methods

This retrospective cross‐sectional study reviewed scans of patients who attended the University Dental Hospital of Sharjah (UDHS). Ethical approval was obtained from the Research Ethics Committee of the University of Sharjah (REC 23‐04‐29‐01‐F). Informed consent was obtained from all patients.

The study included CBCT scans of male and female patients aged 20 years or older, residing in the United Arab Emirates (UAE), who had undergone imaging at UDHS for various clinical indications, provided the scans were of high quality and offered a complete view of the maxillary jaw [9]. CBCT scans were acquired as part of routine clinical care at UDHS for common dental indications, including implant treatment planning, orthodontic assessment, evaluation of impacted teeth, and assessment of suspected dentoalveolar or maxillofacial pathology. All scans were retrospectively retrieved from the institutional archive and were not obtained specifically for research purposes. The exclusion criteria included patients with a history of nasal, nasopharyngeal, paranasal sinus, or adenoidectomy surgery; jaw–facial trauma; congenital nasal anomalies; obvious sinonasal diseases such as chronic rhinosinusitis; patients with congenital craniofacial deformities, for example, cleft lip and palate, maxillary hypoplasia, detectable pharyngeal pathology through image inspection, or pathologies related to the maxillary sinuses were also excluded. Additionally, patients with CBCT images showing supernumerary teeth, bone grafts, large pathologies such as cysts or tumors that alter the anatomy of the study area or prior maxillary sinus surgery were excluded from the study.

The sample size was calculated using G^∗^Power version 3.1.9.7. Based on the parameters reported by Park et al. [10], an effect size of 0.19, a power of 95%, and a significance level of 5% were applied, resulting in a required sample size of 432 scans. To ensure robust analysis, the study included 506 maxillary sinus scans, with volumetric measurements available for 467 CBCT images.

CBCT images were acquired using a Planmeca Viso7 unit (Finland) with exposure settings of 94 kVp, 14 mA, and a 27‐s scan time. The field of view was 18 cm × 20 cm, and a voxel size of 0.2 mm was used. Image analysis was conducted using Romexis software (version 6.2.1.19) [9]. Two trained examiners performed the measurements, with inter‐examiner reliability assessed and discrepancies resolved by consensus. Baseline data were collected from patients’ medical records, including medical and dental history, age, and gender. For age‐group analyses, participants were categorized into age periods of 20–29, 30–39, 40–49, 50–59, and ≥60 years.

The assessment focused on maxillary sinus volume, dental status of the upper jaw, specifically the presence or absence of teeth, and the presence and characteristics of maxillary sinus septa, including their completeness, location, and orientation.

**Figure 1:**
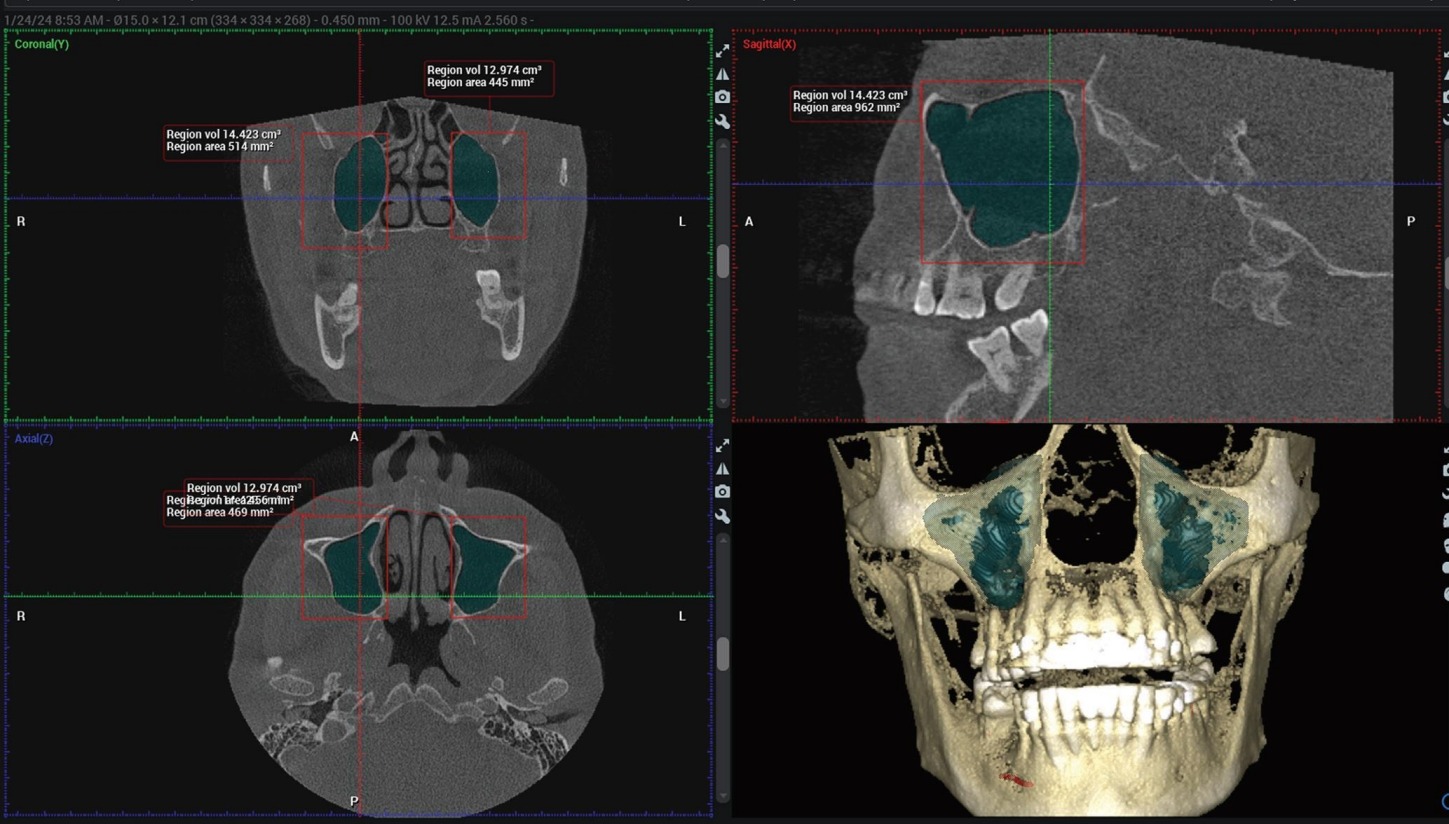
Multiplanar CBCT views in coronal, sagittal, and axial planes, along with a 3D reconstruction, demonstrating bilateral segmentation of the maxillary sinuses highlighted in green using the region‐growing tool.

For maxillary sinus volume measurement, the “region growing tool” in Planmeca Romexis was employed. First, the optimal view where the maxillary sinus was clearly visible in the axial, coronal, and sagittal planes was identified. Using the “measure cube tool,” a 3D bounding box was drawn to encompass the entire sinus within these planes, carefully excluding unrelated areas such as the nasal cavity or artifacts. The segmented area was reviewed across all three views to ensure precision, and manual adjustments were made as needed to refine the boundaries. Once the bounding box was accurately positioned, the “region growing tool” was activated. Segmentation was conducted using a grayscale intensity threshold determined within the CBCT software to differentiate air‐filled sinus cavities from surrounding bone. A seed point was selected by clicking on the darkest area within the cube, initiating automatic segmentation. The software then highlighted the connected region within the specified density range, which was visually displayed in green. When the segmentation was confirmed to accurately represent the sinus cavity, the “volume calculation tool” was used to compute the volume of the segmented region in cubic centimeters, rounded to three decimal places (Figure 1).

The evaluation and localization of maxillary sinus septa were conducted using sagittal, axial, and coronal CBCT images, with panoramic and 3D reconstruction views utilized when necessary. Septa were operationally defined as bony projections extending at least 2 mm into the sinus cavity, without a thickness threshold (Figure 2). The sinus septa were classified according to their anatomical location into anterior, middle, or posterior regions. The anterior region spans from the front wall of the maxillary sinus to the area between the premolars. The middle region lies between the first and second molars, while the posterior region extends from the third molar region to the posterior sinus wall.

Figure 2Septa orientation illustrated with arrows, based on the applied methodology. The coronal septum appears as mediolateral septa in sagittal (a), coronal (b), and axial (c) sections. The sagittal septum is shown in sagittal (d), coronal (e), and axial (f) sections. The transverse septum is displayed in sagittal (g), coronal (h), and axial (i) sections.

**Figure:**
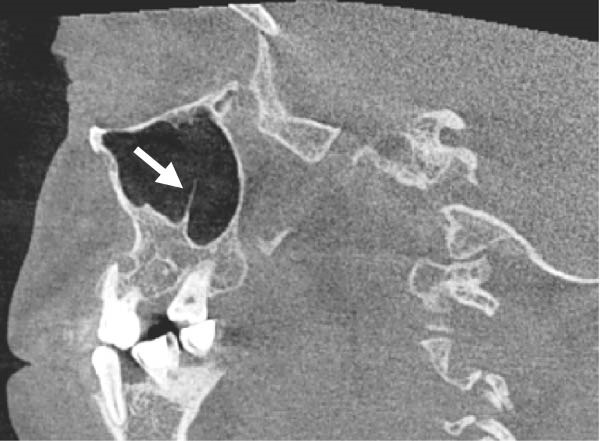
(a)

**Figure:**
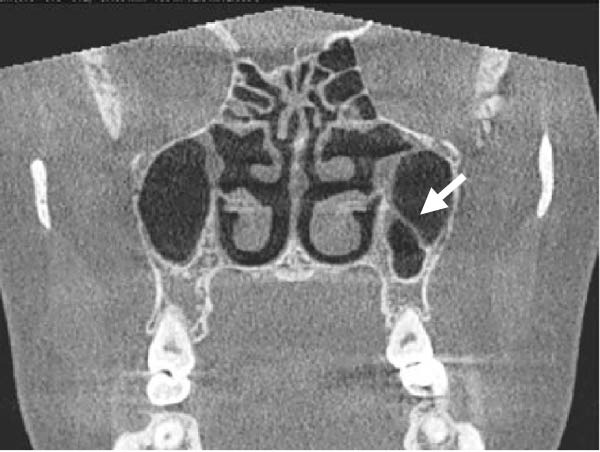
(b)

**Figure:**
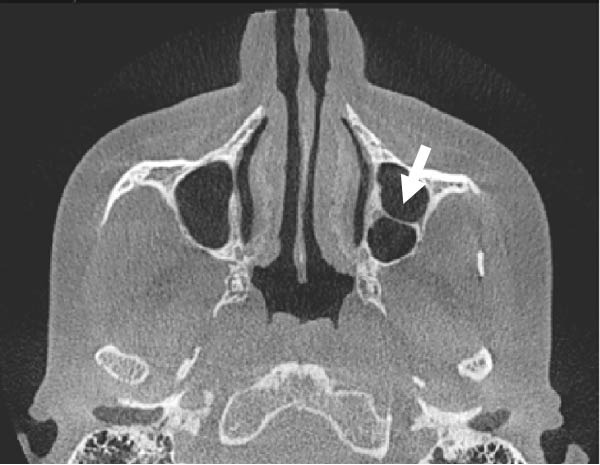
(c)

**Figure:**
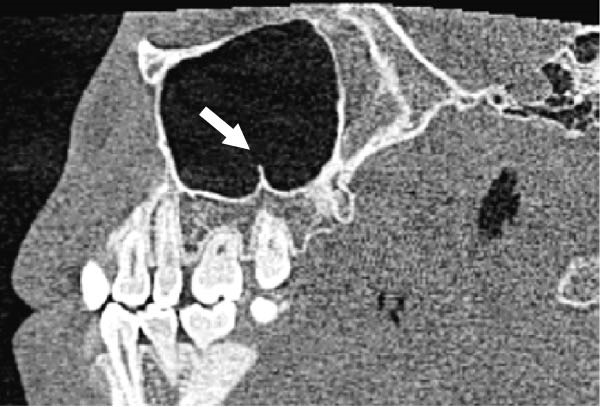
(d)

**Figure:**
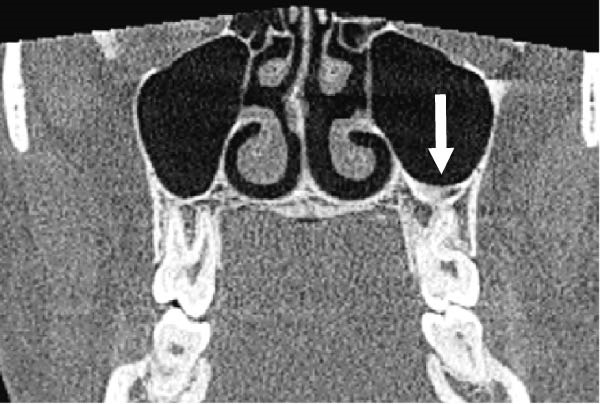
(e)

**Figure:**
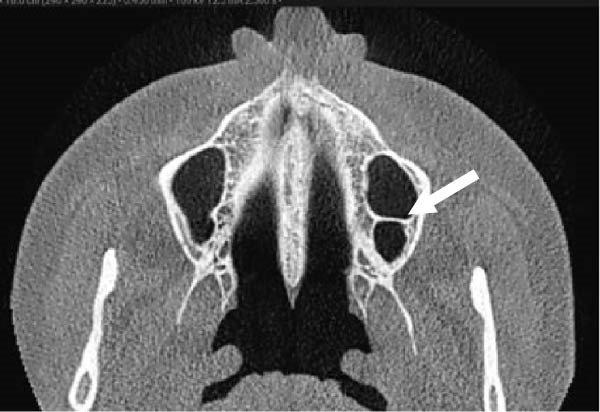
(f)

**Figure:**
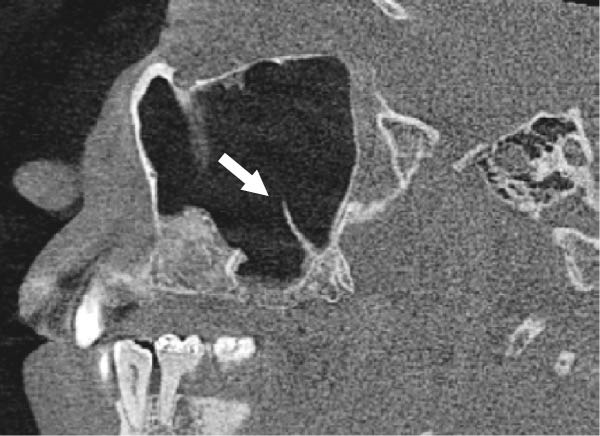
(g)

**Figure:**
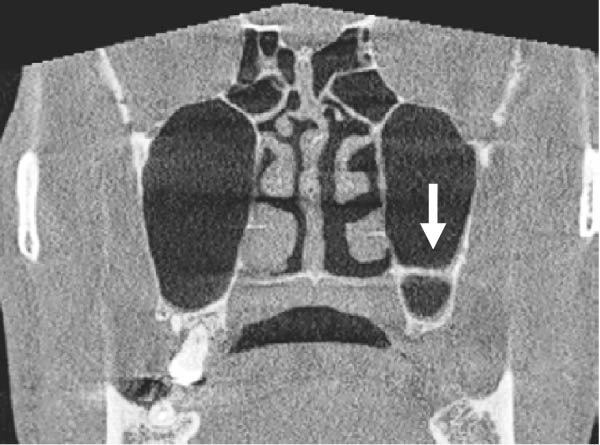
(h)

**Figure:**
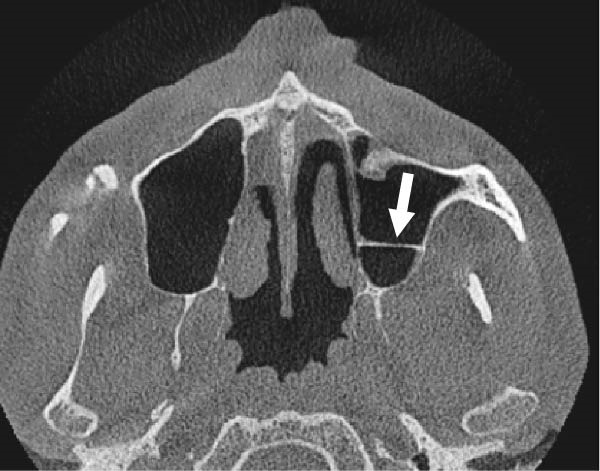
(i)

According to their orientation, maxillary sinus septa were classified into three types: coronal (mediolateral), sagittal, and transverse. The mediolateral septa extended in a buccopalatal direction, connecting the buccal and palatal walls of the sinus. The sagittal septa were aligned parallel to the sagittal plane, extending in an anteroposterior direction. The transverse septa were oriented parallel to the floor of the maxillary sinus.

Moreover, each maxillary sinus septum was recorded as either complete or incomplete. Complete sinus septa arise from one wall of the sinus and then extend until completely reaching an opposing sinus wall. In contrast, incomplete sinus septa do not extend all the way to an opposing sinus wall. The presence of multiple septa was rare. In such instances, the most prominent and longest septum was considered for further analysis.

A random subset comprising 10% (*n* = 25) of the total CBCT scans was reevaluated to test measurement consistency. Intra‐examiner reliability was assessed by repeated measurements performed after a one‐week interval, and inter‐examiner reliability was evaluated by comparing measurements between examiners. Both intra‐ and inter‐examiner reliability were calculated using intraclass correlation coefficients (ICC; two‐way random‐effects model, absolute agreement).

The data were analyzed using SPSS version 28.0 (IBM, Armonk, NY, USA). Descriptive statistics, including frequencies and percentages, were used to summarize patient demographics such as age and sex. To compare two groups, the Student *t*‐test was applied, while differences among multiple groups were assessed using one‐way ANOVA, followed by Bonferroni post‐hoc analysis for pairwise comparisons. The chi‐square test was employed to analyze differences in nonparametric variables. As a robustness assessment, the association between maxillary sinus septa presence and sinus volume was reevaluated using multivariable linear regression after adjustment for age and gender. A *p*‐value of less than 0.05 was considered statistically significant.

## 3. Results

A total of 253 patient CBCT scans were assessed, comprising 116 males (45.8%) and 137 females (54.2%), with a mean age of 39.3 ± 13.6 years (Table 1). These scans encompassed 506 maxillary sinuses, which were evaluated for the presence of septa. Of these, only 467 sinuses were suitable for volumetric analysis, with 231 right and 236 left maxillary sinuses (49.5% and 50.5%, respectively, Table 2). Intra‐examiner reliability was excellent (ICC = 0.93), while inter‐examiner reliability demonstrated similarly high agreement (ICC = 0.91).

**Table 1:** Demographic characteristics of the study population.

Variable	Category	*n* (%)
Age	Mean ± SD (years) 39.30 ± 13.610	—
Gender	Male	116 (45.8)
	Female	137 (54.2)
Dentition status	Dentate	332 (65.6)
	Partially edentulous	162 (32.0)
	Edentulous	12 (2.4)
Maxillary sinus septa	Present	182 (36.0)
	Absent	324 (64.0)

**Table 2:** Maxillary sinus volume according to clinical and anatomical variables.

Variable	Category	*n* (%)	Mean ± SD (cm^3^)	Statistical test	*p*‐Value
Age	(20–29)	139 (29.8)	13.67 ± 3.926	*F* = 0.828	0.508
	(30–39)	125 (26.8)	13.17 ± 5.017		
	(40–49)	109 (23.3)	13.18 ± 5.393		
	(50–59)	42 (9.0)	13.72 ± 5.287		
	≥60	52 (11.1)	12.39 ± 3.471		
Side	Right	231 (49.5)	13.40 ± 5.04	*t* = 0.528	0.598
	Left	236 (50.5)	13.17 ± 4.32		
Gender	Male	214 (45.8)	14.68 ± 5.38	*t* = 5.961	<0.001
	Female	253 (54.2)	12.10 ± 3.62		
Dentition status	Dentate	308 (66.0)	13.42 ± 4.200	*F* = 0.411	0.663
	Partially edentulous	148 (31.7)	13.04 ± 5.577		
	Edentulous	11 (2.4)	12.75 ± 4.666		
Septa presence	Present	165 (35.3)	14.50 ± 4.903	*t* = 4.230	<0.001
	Absent	302 (64.7)	12.62 ± 4.428		

**Figure 3:**
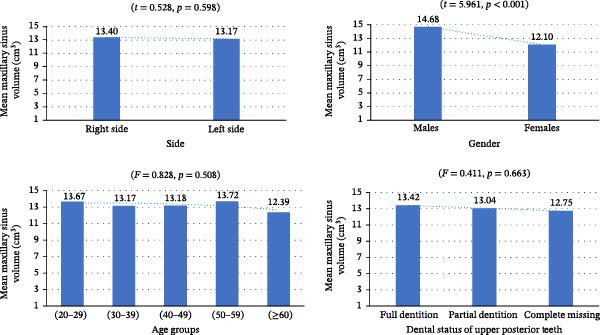
Bar graphs illustrating comparisons of maxillary sinus volume based on side, gender, age groups, and dentition status.

The mean maxillary sinus volume was 13.28 ± 4.68 cm^3^. The average volume on the right side was 13.40 ± 5.04 cm^3^, while on the left it was 13.17 ± 4.32 cm^3^. There was no statistically significant difference between the right and left sides (*t* = 0.528, *p* = 0.598). Males had a significantly larger mean sinus volume (14.68 ± 5.38 cm^3^) compared to females (12.10 ± 3.62 cm^3^) (*t* = 5.961, *p*  < 0.001). No significant differences in sinus volume were found among age groups (*F* = 0.828, *p* = 0.508; Table 2; Figure 3).

The cases were categorized based on dentition status into fully dentate (*n* = 308, 65.95%) with a mean maxillary sinus volume of 13.42 ± 4.200 cm^3^, partially edentulous (*n* = 148, 31.69%) with a volume of 13.04 ± 5.577 cm^3^, and completely edentulous (*n* = 11, 2.35%) with a mean volume of 12.75 ± 4.666 cm^3^. One‐way ANOVA analysis showed no statistically significant difference in maxillary sinus volume among the different dental status groups (*F* = 0.411, *p* = 0.663; Table 2; Figure 3).

**Figure 4:**
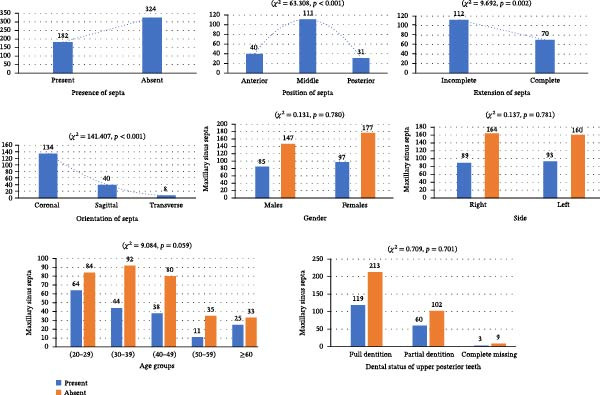
Bar graphs presenting the distribution and comparisons of maxillary sinus septa in terms of their presence, position within the sinus, extension, and orientation, as well as differences across gender, sides, age groups, and dentition status of the maxillary posterior teeth.

Sinus septa were identified in 122 of 253 cases (48.22%), with bilateral presentation in 60 cases (49.18%) and unilateral in 62 cases (50.82%). Overall, septa were present in 182 of the 506 maxillary sinuses examined (35.96%). Of these, 70 cases (14.99%) exhibited complete septa, while 112 cases (23.98%) had incomplete extensions, with a statistically significant difference between these two categories (*χ* ^2^ = 9.692, *p* = 0.002; Figure 4). Multiple septa were rare, occurring in only 2.7% of cases.

Regarding septa location, 40 (21.97%) were located anteriorly, 111 (60.98%) in the middle, and 31 (17.03%) posteriorly within the sinus. This distribution showed a statistically significant difference (*χ* ^2^ = 63.308, *p*  < 0.001; Figure 4).

In terms of orientation, 134 septa (73.62%) were directed coronally, 40 (21.97%) sagittally, and 8 (4.39%) transversely. This distribution showed a statistically significant difference (*χ* ^2^ = 141.407, *p*  < 0.001; Figure 4).

Septa were present in 89 right maxillary sinuses (35.17%) and 93 left maxillary sinuses (36.75%), with no significant side difference (*χ* ^2^ = 0.137, *p* = 0.781). Similarly, septa were observed in 85 males (36.95%) and 97 females (35.40%), showing no statistically significant gender‐based difference (*χ* ^2^ = 0.131, *p* = 0.780; Figure 4).

Based on dentition status, septa were observed in 3 of 12 edentulous cases (25.0%), 60 of 162 partially edentulous cases (37.03%), and 119 of 332 fully dentate cases (35.84%). The difference across these groups was not statistically significant (*χ* ^2^ = 0.709, *p* = 0.701; Figure 4). Septa prevalence did not differ significantly across age groups (*χ* ^2^ = 9.084, *p* = 0.059); however, this borderline value suggests a possible trend toward an age‐related variation that did not reach statistical significance (Figure 4).

**Figure 5:**
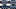
Bar graphs depicting comparisons of maxillary sinus volume based on the presence of sinus septa, as well as their extension, position, and orientation within the maxillary sinuses.

In cases where septa were present, the mean sinus volume was 14.50 ± 4.90 cm^3^, compared to 12.62 ± 4.43 cm^3^ in cases without septa, which was statistically significant (*t* = 4.230, *p*  < 0.001; Table 2). In multivariable linear regression analysis adjusting for age and gender, the presence of maxillary sinus septa remained independently associated with increased maxillary sinus volume. Specifically, sinuses with septa demonstrated a mean volume increase of 1.76 cm^3^ compared with those without septa (*B* = 1.76; 95% CI: 0.92–2.60; *p*  < 0.001). No significant differences in sinus volume were found based on the septa’s position (*F* = 1.031, *p* = 0.359), extent (complete or partial) (*t* = 0.870, *p* = 0.385), or direction (*F* = 0.086, *p* = 0.918; Figure 5).

## 4. Discussion

In this study, detailed analysis of maxillary sinus anatomy revealed several important observations. Sinuses with septa had larger volumes than those without. Septa were frequently observed, most commonly in the middle region of the sinus and typically oriented coronally. Additionally, a slight decline in sinus volume was observed with advancing age, particularly among individuals older than 60 years. Septa were found in 35.96% of the examined maxillary sinuses, within the prevalence range of 6% to 72% reported in previous studies [4, 7, 10–19]. This wide variation may reflect differences in populations, methods, and definitions, such as the minimum height threshold for classification. Factors like ethnicity and dentition status may also contribute [12]. Recognizing these inconsistencies is important for accurate assessment and planning of sinus‐related procedures. Ethnicity was not recorded in the present sample and therefore could not be evaluated directly.

In our study, septa were observed with a nearly equal distribution: bilateral in 49.18% and unilateral in 50.82%. In comparison, previous studies have reported varying distributions, with unilateral septa ranging from 58.3% [14] to 72.31% [15] and bilateral septa from 27.69% [15] to 41.7% [14], while another study reported bilateral septa in 35.25% of cases [18].

Most septa were located in the middle region of the maxillary sinus, typically between the first and second molars, followed by the anterior and posterior regions. In accordance with our results, several previous studies found that the septa were also most commonly located in the middle region [7, 10, 12–15, 18, 19]. This location poses a challenge, as it is often used for lateral window creation during sinus surgery aimed at restoring the first and second molars, which are frequently missing. Surgeons should therefore consider this anatomical feature during preoperative planning. Additionally, these septa can serve as a stable base for dental implants, serving as buttresses that may enhance implant stability [20].

Toprak and Ataç [14] concluded that the middle region’s higher prevalence is likely due to its association with the first and second molars, areas prone to pneumatization changes. In contrast to our findings, a previous study reported that septa were most frequently observed in the anterior region, followed by the posterior, and then the middle region [21].

We found no significant gender differences, in line with other reports [10, 13, 19]. However, some studies have reported conflicting findings regarding gender differences in septa prevalence. While certain studies observed a higher prevalence in males [7, 21], others found a significantly greater occurrence in females [15, 22].

In the current study, no significant differences were found in septa presence between the two sides. Similarly, previous studies reported no statistically significant differences between the right and left sides [7, 10, 16, 18]. Interestingly, some studies reported a higher prevalence of septa on the right side [17, 23]. Conversely, other studies have reported that septa were more frequently found on the left side than on the right [7, 10, 19, 24]. Nevertheless, these studies did not provide a clear explanation for the observed differences in septa location between the two sides.

Complete septa are bony projections that extend across the maxillary sinus, dividing it into compartments, whereas incomplete septa do not span the entire cavity. The present study found that incomplete septa were significantly more common than complete ones. This is in agreement with previous research [6, 12, 15]. Identifying whether a septum is complete or incomplete is essential during preoperative planning for sinus lift procedures, as each type has distinct anatomical and clinical implications. For instance, a complete septum can divide the sinus into separate compartments, potentially impairing mucociliary clearance and requiring a bisectional surgical approach with two lateral windows.

In our study, maxillary sinus septa were most commonly oriented in the coronal plane, corresponding to the buccopalatal direction, followed by the sagittal orientation, while the transverse direction was the least frequent. A similar orientation distribution has been reported in multiple previous studies [10, 12, 13, 15]. Identifying orientation is important for surgical planning to reduce complications such as membrane perforation.

In the current study, there was no difference in the presence of septa among various dentition statuses. Consistent with this finding, several studies concluded that the presence of septa is not significantly related to dental status [10, 15, 16]. In contrast, others found a significantly higher prevalence of septa in edentulous maxillae compared to dentate maxillae [6, 7, 12–14]. This difference was attributed to the tendency of atrophic edentulous regions to develop secondary septa [7]. The comparable presence of maxillary sinus septa across different dentition conditions in the current study may suggest that sinus pneumatization does not consistently follow tooth extraction.

This study found that maxillary sinuses containing septa had significantly greater volumes than those without septa. Notably, a previous study found that the presence of septa was associated with increased sinus width, although no difference in volume was observed [17]. One possible explanation for this key finding in the present study is the presence of an association between sinus size and internal anatomical complexity. Larger sinuses may be more likely to develop internal bony projections as part of developmental remodeling or structural adaptation, reflecting increased surface area and spatial complexity within the sinus cavity. Conversely, the presence of septa may influence the pneumatization process, potentially leading to increased sinus volume. For instance, septa may act as barriers that influence the pneumatization pattern, causing the sinus to expand more in specific directions [14]. Furthermore, septa can act as structural reinforcements within the maxillary sinus. By providing internal support, they may prevent the collapse of the sinus walls, thereby maintaining or even increasing the overall volume of the sinus. This structural function is especially relevant when the sinus is exposed to external pressure or stress [20]. Additionally, the presence of septa is often associated with other anatomical variations in the maxillary sinus, such as the size of the ostium and the thickness of the sinus membrane. Such variations may indirectly affect the measured or perceived sinus volume. For instance, a larger ostium or increased pneumatization in certain regions may contribute to the appearance of a larger sinus volume [17]. From a clinical perspective, the association between septa and larger sinus volumes underscores the importance of careful preoperative imaging assessment, as sinuses with greater volume may exhibit more complex internal anatomy that can influence surgical planning for sinus lift procedures.

Several factors have been reported to influence maxillary sinus volume. Changes in breathing patterns, particularly habitual oral breathing, have been linked to reduced maxillary sinus volume [25]. Similarly, reduced sinus volume has been associated with nasal septum deviation and chronic rhinosinusitis [26]. In addition to functional influences, anatomical variations also play a role. Anatomical factors, including midfacial morphology, have been linked to sinus dimensions, as one study demonstrated a strong correlation between sinus volume and nasal width [27]. Furthermore, one study reported that sinus volume was influenced by sagittal skeletal pattern, noting that individuals with Class III skeletal patterns had larger sinus volumes than those with other classes [28]. These findings highlight the multifactorial nature of sinus volume variation and the need to account for diverse anatomical and physiological factors during clinical evaluation and treatment planning.

In the present study, the mean maxillary sinus volume was ~13 cm^3^, aligning with findings from previous research [1]. Other studies have reported slightly higher mean volumes, such as ~14 cm^3^ [29] and 15 cm^3^ [30]. These discrepancies may be due to differences in age distribution. One study included a younger cohort with a mean age of 22.5 ± 4.32 years [30], while the current sample had a mean age of 39.3 ± 13.6 years. Racial differences have also been proposed as contributing factors in sinus volume variation [31].

Sample type and gender distribution are also important considerations. Several studies have consistently demonstrated that males tend to exhibit larger maxillary sinus volumes than females, which may significantly contribute to variations in reported measurements [30, 32]. Methodological differences further contribute to these inconsistencies. Studies have employed various imaging techniques, most notably CBCT and multi‐detector computed tomography (MDCT), each with distinct resolutions and processing capabilities that may affect volume calculations [26, 30]. Moreover, the method of volume assessment plays a crucial role in measurement variability. Although automated and semiautomated tools aim to improve standardization and efficiency, manual segmentation remains widely used and is highly operator‐dependent, often requiring substantial expertise and time [33].

The present study found no statistically significant differences in sinus volume among age groups, despite a numerical reduction in individuals older than 60. This finding is consistent with previous studies that also reported no significant age‐related differences in sinus volume [1, 3, 29]. However, some studies have reported a reduced maxillary sinus volume in older populations [1, 32, 34, 35]. The observed decline in volume with age may be attributed to physiological changes associated with aging, such as reduced bone density and facial skeletal remodeling [36].

In this study, maxillary sinus volume did not differ significantly across dentition status, whether fully dentate, partially dentate, or edentulous. This finding aligns with the results of previous studies [3, 32]. Thus, edentulism appears to have no substantial effect on sinus dimensions, suggesting that sinus pneumatization does not necessarily continue following tooth loss. Consequently, the loss of vertical bone height in the posterior maxilla following tooth extraction is primarily due to alveolar crest resorption rather than sinus expansion. Similar conclusions have been proposed previously [3, 32, 34].

Our findings confirm that males have larger sinus volumes, consistent with most prior studies [3, 26, 30, 32, 35, 37]. This may be attributed to the generally larger craniofacial structures in males, including the maxillary sinuses. The larger maxillary sinus volumes observed in males may have practical implications for implant planning in the posterior maxilla, potentially influencing sinus lift height and graft volume requirements, provided that other anatomical factors such as posterior maxillary bone height, which were not assessed in this study, are comparable. However, a few studies have reported no significant correlation between maxillary sinus volume and gender [1, 38].

We found no statistically significant difference in maxillary sinus volume between the right and left sides. Similarly, previous studies also reported no significant variation between the two sides [1, 3, 32, 35, 37]. Although studies report no significant difference between right and left maxillary sinus volumes, side‐specific evaluation remains important given possible asymmetry from anatomy, trauma, or surgery.

In this study, CBCT was used as the gold standard imaging method, offering clear visualization of bone and air spaces while avoiding the distortion and superimposition seen in conventional radiographs. Panoramic imaging was excluded due to its high error rate in detecting septa [6, 12].

Volumetric analysis was performed through manual delineation, which is highly dependent on the observer’s skill and accuracy. Inaccuracies in boundary outlining may affect automated volume estimation. Some researchers have suggested estimating volume using mathematical formulas based on width, height, and length. However, this method may be inaccurate due to the maxillary sinus’s complex and irregular shape [39]. Additionally, the current study used a voxel resolution of 0.2 mm for image acquisition. Torres et al. [40] reported no significant differences in linear bone measurements across voxel sizes of 0.2, 0.3, and 0.4 mm.

This study has several limitations that should be considered when interpreting the findings. First, its retrospective cross‐sectional design precluded the systematic inclusion of detailed clinical variables, such as respiratory or metabolic conditions, which may influence craniofacial development. For example, chronic mouth breathing associated with nasal obstruction has been implicated in maxillofacial growth patterns but could not be evaluated in the present analysis. In addition, the absence of comprehensive medical histories limits the interpretation of interindividual variability. Prospective study designs are therefore recommended to better account for such clinical factors, although extended follow‐up periods may be required to achieve adequate sample sizes.

Second, the study was conducted at a single center using CBCT scans acquired with a single imaging unit and a standardized acquisition protocol. While this approach ensured methodological consistency and reduced measurement variability, it may limit the generalizability of the findings to populations imaged using different CBCT systems or acquisition parameters. Multicenter studies incorporating diverse imaging platforms are warranted to confirm and extend these results.

Several anatomical variables reported in the literature to influence maxillary sinus volume, including sagittal skeletal pattern, midface width, and nasal septum deviation, were not assessed in the present study, and therefore could not be evaluated directly. Future studies should incorporate skeletal pattern and nasal airway parameters into multivariable models to further clarify their potential influence on maxillary sinus anatomy. A further limitation of this study is the potential for selection bias, as CBCT examinations were obtained only for specific clinical indications such as implant planning or assessment of impacted teeth. Consequently, the study sample may not be fully representative of the general population.

The underrepresentation of completely edentulous cases limits our ability to draw firm conclusions about maxillary sinus volume and septa presence by dentition status. Additionally, manual segmentation is examiner dependent, representing an additional limitation. Moreover, future studies should incorporate ethnicity as a study variable. This is particularly relevant, as one systematic review has reported a lower prevalence of sinus septa in Asian populations [12]. While our inclusion of individuals from various nationalities enhances generalizability, further subgroup analysis by ethnicity may reveal potential genetic influences, highlighting the value of specifying ethnic background in future research.

## 5. Conclusion

In this study, males exhibited significantly larger maxillary sinus volumes than females, while dentition status had no significant effect. Septa were present in approximately half of the cases, most commonly located in the middle region and predominantly oriented coronally. Their presence did not significantly vary by gender, side, or dentition status. Notably, the presence of septa was linked to greater sinus volume.

These findings underscore the importance of individualized preoperative CBCT assessment of the maxillary sinus for accurate diagnosis and safe planning of implant placement and sinus floor elevation procedures, while also questioning the assumption that sinus volume changes consistently follow tooth loss.

## Author Contributions

Mohammed Amjed Alsaegh made substantial contributions to the conception, design, supervision, interpretation of data, and drafting and revision of the manuscript. Mhd Omar Al Jouhari and Wael Nabil Kharoufah contributed substantially to the methodology, image analysis, statistical analysis, and data validation. Shishir Ram Shetty, Asok Mathew, and Okba Mahmoud contributed to resources, academic support, and critical revision of the manuscript.

## Funding

No funding was received for this manuscript.

## Disclosure

All authors approved the final version.

## Ethics Statement

This retrospective study was approved by the Research Ethics Committee of the University of Sharjah (REC‐23‐04‐29‐01‐F). The study was conducted in accordance with the ethical principles of the Declaration of Helsinki. Informed consent was obtained from all patients.

## Conflicts of Interest

The authors declare no conflicts of interest.

## Data Availability

The data that support the findings of this study are available from the corresponding author upon reasonable request.

## References

[1] Gulec M. , Tassoker M. , Magat G. , Lale B. , Ozcan S. , and Orhan K. , Three-Dimensional Volumetric Analysis of the Maxillary Sinus: A Cone-Beam Computed Tomography Study, Folia Morphologica. (2020) 79, no. 3, 557–562, 10.5603/FM.a2019.0106. 31565786

[2] Yildirim T. T. , Güncü G. N. , Colak M. , Nares S. , and Tözüm T. F. , Evaluation of Maxillary Sinus Septa: A Retrospective Clinical Study With Cone Beam Computerized Tomography (CBCT), European Review for Medical and Pharmacological Sciences. (2017) 21, no. 23, 5306–5314. 29243773 10.26355/eurrev_201712_13912

[3] Schriber M. , Bornstein M. M. , and Suter V. G. A. , Is the Pneumatisation of the Maxillary Sinus Following Tooth Loss a Reality? A Retrospective Analysis Using Cone Beam Computed Tomography and a Customised Software Program, Clinical Oral Investigations. (2019) 23, no. 3, 1349–1358, 10.1007/s00784-018-2552-5, 2-s2.0-85049978203. 30014166

[4] Assari A. , Alotaibi N. , Alajaji M. A. , Alqarni A. , and Ali Alarishi M. , Characteristics of Maxillary Sinus Septa: A Cone-Beam Computed Tomography Evaluation, International Journal of Dentistry. (2022) 2022, no. 1, 10.1155/2022/2050257, 2050257. 36249727 PMC9556247

[5] Underwood A. S. , An Inquiry Into the Anatomy and Pathology of the Maxillary Sinus, Journal of Anatomy and Physiology. (1910) 44, no. Pt 4, 354–369. 17232856 PMC1289237

[6] Krennmair G. , Ulm C. , and Lugmayr H. , Maxillary Sinus Septa: Incidence, Morphology and Clinical Implications, Journal of Cranio-Maxillofacial Surgery. (1997) 25, no. 5, 261–265, 10.1016/S1010-5182(97)80063-7, 2-s2.0-0030693673. 9368861

[7] Kim M. J. , Jung U. W. , and Kim C. S. , et al.Maxillary Sinus Septa: Prevalence, Height, Location, and Morphology. A Reformatted Computed Tomography Scan Analysis, Journal of Periodontology. (2006) 77, no. 5, 903–908, 10.1902/jop.2006.050247, 2-s2.0-33746001057. 16671885

[8] Boyne P. J. and James R. A. , Grafting of the Maxillary Sinus Floor With Autogenous Marrow and Bone, Journal of Oral Surgery. (1980) 38, no. 8, 613–616. 6993637

[9] Alsaegh M. A. , Almulla O. , Almutairi N. , Alkandari B. , and Altourah A. , The Relationship Between the Thickness of the Buccal and Palatal Cortical Bone Surrounding the Maxillary Third Molars and the Corresponding Dimensions of the Maxillary Tuberosity: A Retrospective Cross-Sectional Study, BMC Oral Health. (2025) 25, no. 1, 10.1186/s12903-025-06596-w, 1282. 40739227 PMC12312287

[10] Park Y.-B. , Jeon H.-S. , Shim J.-S. , Lee K.-W. , and Moon H.-S. , Analysis of the Anatomy of the Maxillary Sinus Septum Using 3-Dimensional Computed Tomography, Journal of Oral and Maxillofacial Surgery. (2011) 69, no. 4, 1070–1078, 10.1016/j.joms.2010.07.020, 2-s2.0-79953226965. 21255895

[11] Jung J. W. , Song K. H. , and Lee S. K. , et al.The Clinical Study of Maxillary Sinus Septa Used in Panorama, CT, Journal of the Korean Association of Oral and Maxillofacial Surgeons. (2008) 34, no. 3, 319–324.

[12] Pommer B. , Ulm C. , Lorenzoni M. , Palmer R. , Watzek G. , and Zechner W. , Prevalence, Location and Morphology of Maxillary Sinus Septa: Systematic Review and Meta-Analysis, Journal of Clinical Periodontology. (2012) 39, no. 8, 769–773, 10.1111/j.1600-051X.2012.01897.x, 2-s2.0-84863779554. 22624862

[13] Hungerbühler A. , Rostetter C. , Lübbers H.-T. , Rücker M. , and Stadlinger B. , Anatomical Characteristics of Maxillary Sinus Septa Visualized by Cone Beam Computed Tomography, International Journal of Oral and Maxillofacial Surgery. (2019) 48, no. 3, 382–387, 10.1016/j.ijom.2018.09.009, 2-s2.0-85055093501. 30360991

[14] Toprak M. E. and Ataç M. S. , Maxillary Sinus Septa and Anatomical Correlation With the Dentition Type of Sinus Region: A Cone Beam Computed Tomographic Study, British Journal of Oral and Maxillofacial Surgery. (2021) 59, no. 4, 419–424, 10.1016/j.bjoms.2020.08.038. 33714626

[15] Benjaphalakron N. , Jansisyanont P. , Chuenchompoonut V. , and Kiattavorncharoen S. , Evaluation of the Maxillary Sinus Anatomical Variations Related to Maxillary Sinus Augmentation Using Cone Beam Computed Tomography Images, Journal of Oral and Maxillofacial Surgery, Medicine, and Pathology. (2021) 33, no. 1, 18–25, 10.1016/j.ajoms.2020.07.001.

[16] Schiller L. A. , Barbu H. M. , Iancu S. A. , and Brad S. , Incidence, Size and Orientation of Maxillary Sinus Septa—A Retrospective Clinical Study, Journal of Clinical Medicine. (2022) 11, no. 9, 10.3390/jcm11092393, 2393. 35566519 PMC9103037

[17] Aşantoğrol F. and Coşgunarslan A. , The Effect of Anatomical Variations of the Sinonasal Region on Maxillary Sinus Volume and Dimensions: A Three-Dimensional Study, Brazilian Journal of Otorhinolaryngology. (2022) 88, no. suppl. 1, S118–S127, 10.1016/j.bjorl.2021.05.001. 34053909 PMC9734263

[18] Mirdad A. , Alaqeely R. , Ajlan S. , Aldosimani M. A. , and Ashri N. , Incidence of Maxillary Sinus Septa in the Saudi Population, BMC Medical Imaging. (2023) 23, no. 1, 10.1186/s12880-023-00980-0, 23. 36739395 PMC9898957

[19] Altayar B. A. , Al-Tayar B. , and Lin W. , et al.Cone-Beam Computed Tomographic Analysis of Maxillary Sinus Septa Among Yemeni Population: A Cross-Sectional Study, BMC Oral Health. (2023) 23, no. 1, 10.1186/s12903-023-03124-6, 466. 37422645 PMC10329384

[20] Gatilo I. , Sirak S. , and Lenev V. , et al.Internal Septa of the Maxillary Sinus and Their Significance in the Planning of Endodontic Interventions, Sinus Lifting and Dental Implantation Using a Collagen Matrix Scaffold, Endodontics Today. (2024) 22, no. 4, 388–397, 10.36377/ET-0044.

[21] Hong K. L. , Wong R. C. W. , Lim A. A. T. , Loh F. C. , Yeo J. F. , and Islam I. , Cone Beam Computed Tomographic Evaluation of the Maxillary Sinus Septa and Location of Blood Vessels at the Lateral Maxillary Sinus Wall in a Sample of the Singaporean Population, Journal of Oral and Maxillofacial Surgery, Medicine, and Pathology. (2017) 29, no. 1, 39–44, 10.1016/j.ajoms.2016.09.005, 2-s2.0-84991278155.

[22] Asan M. F. , Castelino R. L. , Babu S. G. , and Darwin D. , Anatomical Variations of the Maxillary Sinus – A Cone Beam Computed Tomography Study, Acta Medica Bulgarica. (2022) 49, no. 3, 33–37, 10.2478/amb-2022-0027.

[23] Koymen R. , Gocmen-Mas N. , Karacayli U. , Ortakoglu K. , Ozen T. , and Yazici A. C. , Anatomic Evaluation of Maxillary Sinus Septa: Surgery and Radiology, Clinical Anatomy. (2009) 22, no. 5, 563–570, 10.1002/ca.20813, 2-s2.0-67650370132. 19484797

[24] Alec M. , Smektała T. , Trybek G. , and Sporniak-Tutak K. , Maxillary Sinus Septa: Prevalence, Morphology, Diagnostics and Implantological Implications. Systematic Review, Folia Morphologica. (2013) 73, 259–266.

[25] Agacayak K. S. , Gulsun B. , Koparal M. , Atalay Y. , Aksoy O. , and Adiguzel O. , Alterations in Maxillary Sinus Volume Among Oral and Nasal Breathers, Medical Science Monitor: International Medical Journal of Experimental and Clinical Research. (2015) 21, 18–26, 10.12659/MSM.891371, 2-s2.0-84920183198. 25553770 PMC4289671

[26] Taneja A. , Malhotra A. , and Chandak S. , et al.Volumetric Analysis of Maxillary Sinus and Assessment of Various Sinonasal Anatomic Variants on Multi-Detector Computed Tomography (MDCT) and Their Association With Chronic Rhinosinusitis, Journal of Clinical Imaging Science. (2024) 14, 10.25259/JCIS_124_2024, 44. 39639883 PMC11618756

[27] Alhazmi A. , Association Between Maxillary Sinus Dimensions and Midface Width: 2-D and 3-D Volumetric Cone-Beam Computed Tomography Cross-Sectional Study, The Journal of Contemporary Dental Practice. (2020) 21, no. 3, 317–321, 10.5005/jp-journals-10024-2725. 32434981

[28] Lessa A. M. G. , Oliveira V. S. , and Costa R. B. A. , et al.Anatomical Study of the Maxillary Sinus: Which Characteristics Can Influence its Volume?, Surgical and Radiologic Anatomy. (2023) 45, no. 1, 81–87, 10.1007/s00276-022-03055-x. 36474022

[29] Rinaldi F. , Piattelli M. , Angiolani F. , Bernardi S. , Rastelli E. , and Varvara G. , Volumetric Evaluation of Maxillary Sinuses Using CBCTs: Radiographic Study, Italian Journal of Anatomy and Embryology. (2023) 127, no. 2, 47–50, 10.36253/ijae-14681.

[30] Dinç K. and İçöz D. , Maxillary Sinus Volume Changes in Individuals With Different Craniofacial Skeletal Patterns: CBCT Study, BMC Oral Health. (2024) 24, no. 1, 1516. 39702251 10.1186/s12903-024-05341-z PMC11661000

[31] Fernandes C. L. , Volumetric Analysis of Maxillary Sinuses of Zulu and European Crania by Helical, Multislice Computed Tomography, Journal of Laryngology and Otology. (2004) 118, no. 11, 877–881. 15638975 10.1258/0022215042703705

[32] Bornstein M. M. , Ho J. K. C. , Yeung A. W. K. , Tanaka R. , Li J. Q. , and Jacobs R. , A Retrospective Evaluation of Factors Influencing the Volume of Healthy Maxillary Sinuses Based on CBCT Imaging, The International Journal of Periodontics & Restorative Dentistry. (2019) 39, no. 2, 187–193, 10.11607/prd.3722, 2-s2.0-85061998784. 30794254

[33] Giacomini G. , Pavan A. L. M. , and Altemani J. M. C. , et al.Computed Tomography-Based Volumetric Tool for Standardized Measurement of the Maxillary Sinus, PLoS ONE. (2018) 13, no. 1, 10.1371/journal.pone.0190770, 2-s2.0-85040039288. PMC5755892 29304130

[34] Velasco-Torres M. , Padial-Molina M. , and Avila-Ortiz G. , et al.Maxillary Sinus Dimensions Decrease as Age and Tooth Loss Increase, Implant Dentistry. (2017) 26, no. 2, 288–295, 10.1097/ID.0000000000000551, 2-s2.0-85010877232. 28125519

[35] Belgin C. A. , Colak M. , Adiguzel O. , Akkus Z. , and Orhan K. , Three-Dimensional Evaluation of Maxillary Sinus Volume in Different Age and Sex Groups Using CBCT, European Archives of Oto-Rhino-Laryngology. (2019) 276, no. 5, 1493–1499, 10.1007/s00405-019-05383-y, 2-s2.0-85063051569. 30879193

[36] Iturralde-Garrote A. , Sanz J. L. , Forner L. , Melo M. , and Puig-Herreros C. , Volumetric Changes of the Paranasal Sinuses With Age: A Systematic Review, Journal of Clinical Medicine. (2023) 12, no. 10, 2023–3355, 10.3390/jcm12103355. 37240460 PMC10219259

[37] Betancourt A. B. R. , Somoza L. J. M. , and Mesa C. R. , et al.Relationship of Maxillary Sinus Volume and Nasal Septum Deviation: A Cone Beam Computed Tomography Study, Diagnostics. 14, no. 6, 2024–2647. 38535067 10.3390/diagnostics14060647 PMC10969206

[38] Saccucci M. , Cipriani F. , and Carderi S. , et al.Gender Assessment Through Three-Dimensional Analysis of Maxillary Sinuses by Means of Cone Beam Computed Tomography, European Review for Medical and Pharmacological Sciences. (2015) 19, no. 2, 185–193. 25683929

[39] Monsour P. A. and Dudhia R. , Implant Radiography and Radiology, Australian Dental Journal. (2008) 53, no. s1, S11–S25, 10.1111/j.1834-7819.2008.00037.x, 2-s2.0-44349096297. 18498579

[40] Torres M. G. , Campos P. S. , Segundo N. P. , Navarro M. , and Crusoé-Rebello I. , Accuracy of Linear Measurements in Cone Beam Computed Tomography With Different Voxel Sizes, Implant Dentistry. (2012) 21, no. 2, 150–155, 10.1097/ID.0b013e31824bf93c, 2-s2.0-84859162468. 22382754

